# Arterial tortuosity syndrome causing recurrent transient ischemic attacks in young adult: a case report

**DOI:** 10.1186/s12883-021-02458-4

**Published:** 2021-11-30

**Authors:** Stefano Cotti Piccinelli, Enrico Premi, Sonia Bonacina, Nicola Gilberti, Veronica Vergani, Massimo Gamba, Raffaella Spezi, Ilenia Delrio, Michaël Bernier, Angelo Costa, Marco Ravanelli, Marina Colombi, Roberto Gasparotti, Alessandro Padovani, Mauro Magoni

**Affiliations:** 1 Neurology Unit, Department of Clinical and Experimental Sciences, University of Brescia, Pz.le Spedali Civili 1, 25100 Brescia, Italy; 2 Stroke Unit, Unità Operativa Neurologia Vascolare, ASST “Spedali Civili”, Spedali Civili, Brescia, Italy; 3 Martinos Center for Biomedical Imaging, Harvard Medical School, Massachusetts General Hospital, Charlestown, MA USA; 4 Radiology Unit, Department of Medical-Surgical Specialties, Radiological Sciences and Public Health, University of Brescia, Brescia, Italy; 5 Division of Biology and Genetics, Department of Molecular and Translational Medicine, University of Brescia, Brescia, Italy; 6 Neuroradiology Unit, Department of Medical-Surgical Specialties, Radiological Sciences and Public Health, University of Brescia, Brescia, Italy

**Keywords:** Arterial tortuosity syndrome, Transient ischemic attack, Thrombolysis

## Abstract

**Background:**

Arterial Tortuosity Syndrome (ATS) is a rare autosomal recessive disorder characterized by elongated and tortuous arteries. Although ATS showed a significant clinical and pathophysiological overlap with other syndromes involving connective tissues, only few cases of cerebrovascular events related to this syndrome have been described so far.

**Case presentation:**

We report the case of a 33-years-old male diagnosed with ATS since childhood, that experienced three sudden episodes of expressive aphasia and right hemiparesis with spontaneous resolution. He was treated with recombinant tissue plasminogen activator (r-TPA) at a dosage of 0.9 mg/kg with a complete recovery. Brain Magnetic Resonance Imaging (MRI) showed the absence of acute ischemic lesions and the patient was diagnosed with recurrent transient ischemic attacks (TIA).

Intracranial and supra-aortic trunks Magnetic Resonance Angiography (MRA) and Angio-CT scan of the thoracic and abdominal aorta showed marked vessel tortuosity without stenosis.

To our knowledge, this is the first reported case of an ATS patient with TIA in young age that was treated with intravenous thrombolysis with recombinant plasminogen activator.

**Conclusion:**

Our report strengthens the relationship between ATS and juvenile cerebrovascular events, suggesting that an extensive study of body vessels in order to detect potential stenoses or occlusions in these cases is needed.

The greater predisposition to cerebrovascular events in ATS could benefit from a more aggressive primary and secondary prevention therapy.

## Background

Arterial Tortuosity Syndrome (ATS) is a rare, autosomal recessive connective tissue disorder characterized by elongated and tortuous large and medium-sized arteries and a propensity to aneurysm formation, vascular dissection and stenosis of the pulmonary arteries.

ATS is caused by loss-of-function mutations in the *SLC2A10* gene encoding the facilitative glucose transporter GLUT10 [1]. GLUT10 intracellular location remains uncertain and consequently also the exact pathogenesis of this syndrome. The main hypothesis is that *SLC2A10* encodes a transporter of dehydroxyascorbic acid that may be present both over mitochondrial membranes and endoplasmic reticulum. Ascorbic acid is crucial for elastin and collagen maturation working as a hydroxylation cofactor for both prolyl and lysyl residues [2]. The reduction of hydroxylation of these residues appears to lead to impairment of vessel walls due to the perturbation of the transforming growth factor beta pathway caused by GLUT10 deficiency [3, 4]. Large vessel biopsies revealed reduction of smooth elastic lamella with highly disorganized, thicker and fragmented elastic fibers in vascular tissue [1].

Clinical onset usually occurs in infancy or early childhood. The most common manifestations are cardiovascular and include right ventricular hypertension, acute respiratory symptoms, ventricular hypertrophy and cardiac failure. Aneurysm formation and arterial dissection are frequent and may involve cerebrovascular circulation [1]. Other manifestations are dysmorphic features, keratoconus, hyperextensible skin, joint hypermobility, skeletal abnormalities, and generalized hypotonia [1].

Although ATS shows a significant clinical and pathophysiological overlap with other diseases involving connective tissues, only few cases of cerebrovascular events related to this syndrome have been described so far [1, 5, 6].

Despite the description of several cases to date, clinical spectrum and natural history are still not completely understood and therefore, clinical management relies mainly on expert opinion [1].

## Case presentation

We report the case of a 33-year-old Italian male born from consanguineous parents and diagnosed with ATS since adolescence carrying the homozygous c.1334delG (p.Gly445-Glufs*40) *SLC2A10* pathogenic variant [1, 7].

He suffered a cardiac arrest at delivery. Reanimation was successfully performed, and no brain damages occurred. He had normal psycho-physical development. During childhood and adolescence his major alterations were tortuosity, dilatation, and elongation of the main arteries and an increase carotid bifurcation thickness of intima and media, associated with vomiting, failure to thrive, dyspnea, diaphoresis, arm and abdominal pain, and fainting. He also suffered recurrent fever, bronchitis, and pneumonia successfully treated with antibiotic therapy. At 19 years, aortic sinus dilation (48 mm) with a moderate aortic valve incompetence were detected by aortic angiography and computed tomography (CT). Doppler ultrasonography showed extreme narrowing and tortuosity of the arteries. Therefore, the patient underwent surgery by replacing the ascending aorta according to Tyrone-Davis procedure with Vascutek No.28 prosthesis and aortic valve sparing [7, 8]. He also suffered from autoimmune chronic thyroiditis, treated with Levotiroxine 150 mcg daily, and Gilbert’ syndrome. He received cornea transplantation in left eye for corneal ectasia.

At 33 years, while he was at home, he experienced an episode characterized by expressive aphasia and right hemiparesis. Symptoms were described by family members lasting about 15 min with spontaneous resolution. While the patient was being transported to the hospital, he suffered a new episode with similar characteristics, lasting about 10 min. A further episode was observed once the patients arrived at the emergency room. Neurological evaluation showed central deficit of the VII right cranial nerve, mild right hemiparesis, mild expressive aphasia with some naming deficit and mild dysarthria (National Institute of Health Stroke Scale, NIHSS = 6). Brain CT showed no hyperacute signs of ischemia. Glucose, coagulation and blood cell counts were normal. After 20 min, neurological picture began to improve spontaneously, but without a complete recovery. In accordance with the international stroke guidelines, the patient was admitted to the Stroke Unit and intravenous fibrinolytic therapy with recombinant tissue plasminogen activator (rTPA) at a dosage of 0.9 mg/kg was administered.

After 1 h, the neurological examination was unremarkable. No signs of ischemia or bleeding complications were evident at brain CT after 24 h. Electroencephalogram, urine analysis, electrolytes, coagulation and routine screening for juvenile stroke including thyroid ormones were normal.

**Fig. 1:**
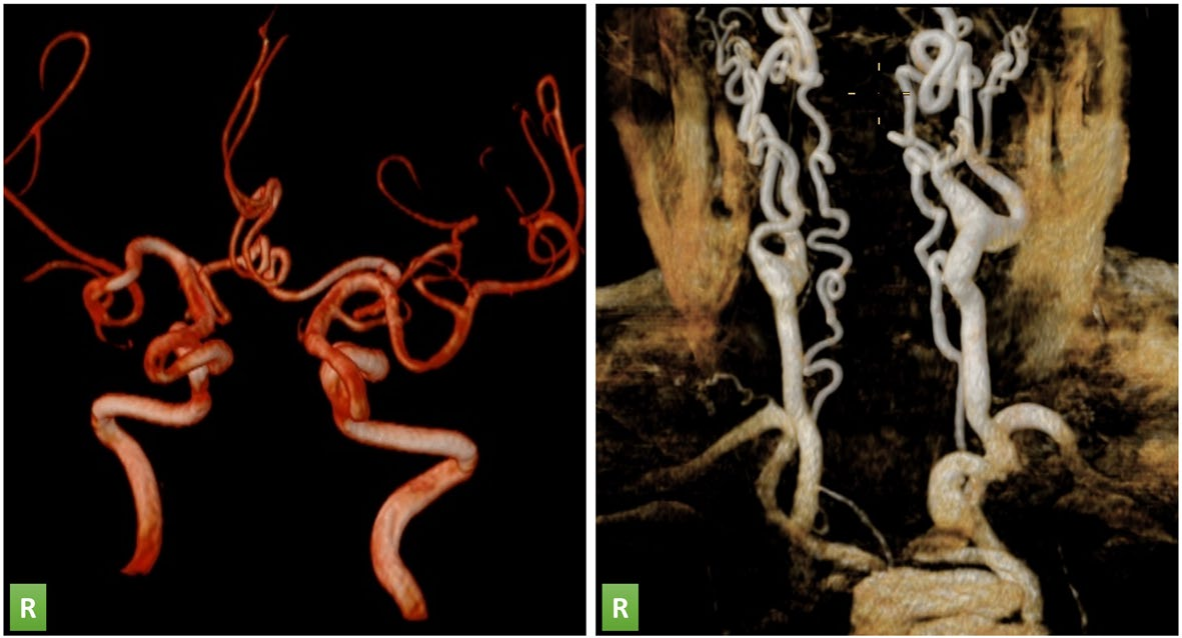
Three-dimension reconstruction of Intracranial (on the left) and supra-aortic (on the right) trunks from Magnetic Resonance Angiography (MRA) of the ATS patient, showing a marked tortuosity of the vessels, in the absence of dissection, aneurysmal lesions or hemodynamically significant stenoses. R: right

3 Tesla brain magnetic resonance imaging (MRI) T1, T2 and FLAIR sequence showed the absence of acute ischemic lesions. Intracranial and supra-aortic trunks magnetic resonance angiography (MRA) showed a marked tortuosity of the vessels, in the absence of dissection, aneurysmal lesions or hemodynamically significant stenoses (Fig. 1)

Transesophageal echocardiography showed a moderate hypertrophy of the left ventricle with signs of diastolic dysfunction. There were no signs of patent foramen ovalis or shunt. Blood pressure values were monitored but were within normal limits.

**Fig. 2:**
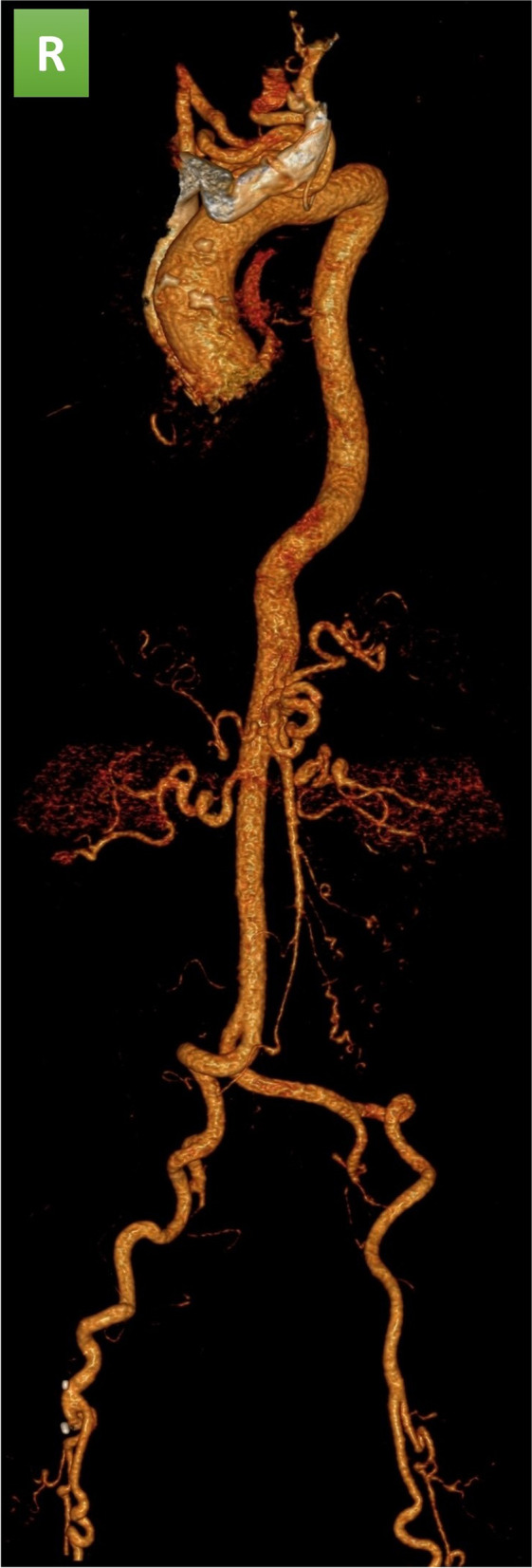
Three-dimension reconstruction of Angio-CT scan of the thoracic and abdominal aorta of the ATS patient, showing tortuosity of thoracic and abdominal vessel without stenosis. R: right

Angio-CT scan of the thoracic and abdominal aorta showed tortuosity of thoracic and abdominal vessel without any stenosis (Fig. 2).

The patient was diagnosed with recurrent transient ischemic attacks (TIA) and discharged with Aspirin 100 mg daily, with periodic follow-up and blood pressure monitoring indications.

At 3 months follow-up the patient was still asymptomatic.

## Discussion and conclusion

Despite few cases published, our report helps to strengthen the relationship between ATS and juvenile cerebrovascular events. In a recent review of the published cases, *Beyens* et Al. reported two cases of juvenile stroke in ATS patients, one in association with intraparenchymal hemorrhage and one of unknown etiology resulting in left hemiparesis [1]. *Cartwright* et Al. reported a case of a 14 y.o female who presented with left basal ganglia ischemic stroke, but no others additional stroke risk factors were identified [5].

Since juvenile cerebrovascular events in ATS patients are rare, the mechanism by which ATS leads to cerebrovascular manifestations has not been established yet. Several hypotheses have been proposed. It may involve alterations of the endothelium and subsequent arterial thrombosis, vessel stenosis evolving to occlusion and infarction or dissection of affected arteries.

In this case, none of these anomalies were evident in intracranial vessels and supra-aortic trunks.

Since the patient carried a prosthesis of the ascending aorta, albeit with aortic valve sparing, an embolic genesis of the stroke cannot be excluded. However, imaging of the thoracic and abdominal vessels showed no significant abnormalities, except for the marked tortuosity of the vessels.

Thus, the origin of the recurrent transient ischemic attacks remains cryptogenic.

In consideration of the marked vessel alterations, patients suffering from this syndrome should be monitored closely for cerebrovascular risk factors and potential recurrences.

At diagnosis, imaging of cerebral and epiaortic vessels may be recommended in order to identify stenosis or aneurysms.

Furthermore, to our knowledge, this is the first reported case of an ATS patient treated with intravenous thrombolysis with rTPA. This treatment appeared to be effective and safe in the subject here described, supporting further future use in this type of patients.

The potential increased predisposition towards cerebrovascular events in ATS could suggest a benefit from an antiplatelet therapy, in these subjects, also as primary prevention strategy.

However, these preliminary findings could deserve further confirmations with studies on larger cohort of subjects with ATS.

### Patient perspective

The patient and the patient’s family were aware of the genetic diagnosis made years earlier but did not think it could generate any risk of cerebral ischemic events.

After hospitalization, the patient returned to a totally normal life and resumed work.

It was followed up by both clinical and telephone follow-up and, at the time of writing, no new episodes were presented.

The prospect that other similar events could occur worried the patient, but the patient was informed about what the early signs of cerebral ischemic events may be and about the need to be prompt in their recognition in order to obtain acute treatment.

## Data Availability

Data presented are available on request from the corresponding author.

## Abbreviations

ATS: Arterial tortuosity syndrome
CT: Computed tomography
MRI: Magnetic resonance imaging
r-TPA: Recombinant tissue plasminogen activator
